# Effect of knee alignment on the quadriceps femoris muscularity: Cross-sectional comparison of trained versus untrained individuals in both sexes

**DOI:** 10.1371/journal.pone.0183148

**Published:** 2017-08-14

**Authors:** Ryoichi Ema, Taku Wakahara, Kuniaki Hirayama, Yasuo Kawakami

**Affiliations:** 1 Graduate School of Engineering and Science, Shibaura Institute of Technology, Saitama, Japan; 2 Research Fellow of Japan Society for the Promotion of Science, Tokyo, Japan; 3 Faculty of Health & Sports Science, Doshisha University, Kyoto, Japan; 4 Faculty of Sport Sciences, Waseda University, Saitama, Japan; Semmelweis Egyetem, HUNGARY

## Abstract

Knee alignment is suggested to be a factor affecting each quadriceps femoris muscle size, and knee alignment such as Q-angle differs between men and women. Also, training can induce inhomogeneous hypertrophy among the quadriceps femoris, thereby leading to different component characteristics of the muscles. If Q-angle is a major determinant of the quadriceps femoris muscularity, it is hypothesized that the sex-related difference in the quadriceps femoris muscularity, if any, is further highlighted in trained individuals, being associated with Q-angle. We tested this hypothesis. Magnetic resonance images of the right thigh were obtained from 26 varsity rowers as trained subjects (13 for each sex) and 34 untrained individuals as controls (17 for each sex). From the images, muscle volume of each constituent of the quadriceps femoris (vastus lateralis, VL; medialis, VM; intermedius; rectus femoris) was determined. The Q-angle was measured during quiet bilateral standing with hand support as needed. Percent volume of VM to the total quadriceps femoris was greater in female rowers than male rowers and female controls, and that of VL was greater in male rowers than male controls. There were no correlations between Q-angle and percent muscle volume in any muscles regardless of rowing experience or sex. The current study revealed that well-trained rowers have sex-related quadriceps femoris muscularity but no significant correlations between percent muscle volume in any muscles and Q-angle. Our findings suggest that Q-angle is not a major determinant of the quadriceps femoris muscularity in either well-trained or untrained individuals.

## Introduction

The quadriceps femoris, which plays an important role during exercise performance [1] and daily activities [2], is composed of the vastus lateralis (VL), vastus medialis (VM), vastus intermedius (VI) and rectus femoris (RF). The four muscles have different anatomical features such as the number of joints that the muscles cross and fiber orientations. The different features should be associated with functional differences among muscles. For example, muscle activation during multi-joint leg extensions (simultaneous extensions of knee and hip joints) was different among monoarticular VL or VM and biarticular RF [3]. The VM, which is the only medial component of the four muscles, is considered to counterbalance the lateral pull of VL to stabilize the patella [4]. These anatomical and functional differences may be related to the quantitative characteristics of individual muscles. In untrained men, percent muscle volume to the total quadriceps femoris is largest in VL (32.6%), followed by VI (27.8%), VM (25.0%) and RF (14.8%) [5]. In contrast, there is to some extent inter-individual variability of the constituents (the coefficient of variations [CV] of the percent volume among the subjects were ~11.5% [5]). Clarification of the possible factors that relate to the variability could provide better understanding of functional role of individual muscles.

Knee alignment has been suggested as possibly contributing to the relative size of each constituent of the quadriceps femoris [6,7]. The anatomical cross-sectional areas (ACSAs) of VL, VM and VI were reportedly greater in untrained men with a larger quadriceps femoris angle (Q-angle) compared to ones who had a smaller Q-angle, whereas the ACSA of the RF was similar for the two different Q-angle groups [6]. The percent ACSAs of VL and RF to the total quadriceps femoris were greater in the untrained men with genu valgum (i.e., large Q-angle [8]) than with genu varum, and vice versa for that of VM [7]. Although there are some discrepancies between the previous studies [6,7], the findings suggest that Q-angle affects each muscle size of the quadriceps femoris in untrained individuals: there may be an association between Q-angle and the quadriceps femoris muscularity. If so, component characteristics of the quadriceps femoris may differ between untrained men and women due to a sex difference in Q-angle [9].

Training status may also affect quadriceps femoris muscularity. A number of studies demonstrated that the magnitude of training-induced increase in muscle size (hypertrophy) was inhomogeneous among the four muscles of the quadriceps femoris [10–12]. Therefore, trained individuals may display differential proportional development of the component muscles within the quadriceps femoris when compared to untrained individuals. Indeed, the percent muscle volume of VL to the total quadriceps femoris was higher in the well-trained male rowers [5] and cyclists [12] than untrained men.

The purpose of the current study was to examine the effect of knee alignment on the quadriceps femoris muscularity. If Q-angle is a major determinant of the muscularity, it is possible that the sex-related difference in the quadriceps femoris muscularity, if any, is further highlighted in trained individuals, being associated with Q-angle. To this end, we compared the quadriceps femoris muscularity among trained and untrained men and women and to examine the relationships between component characteristics of the quadriceps femoris and Q-angle. We recruited well-trained rowers as trained individuals, because (1) most of the lower-extremity motions involved in rowing are repetitive multi-joint leg extensions and (2) the main contributor to leg extension power is the quadriceps femoris [13]. Therefore, compared to other sport activities in which several different motions are involved, the effect of training on the association of the muscularity of the quadriceps femoris with Q-angle would likely be much more straightforward.

## Materials and methods

### Subjects

Twenty-six well-trained male and female varsity rowers (n = 13 for each sex, male; 21 ± 1 yr, 177 ± 7 cm, 76 ± 8 kg; female, 20 ± 1 yr, 168 ± 7 cm, 63 ± 9 kg) and 34 untrained university students (controls, n = 17 for each sex, male; 22 ± 2 yr, 170 ± 5 cm, 65 ± 9 kg; female, 22 ± 2 yr, 161 ± 5 cm, 53 ± 5 kg) participated in this study. No subjects had knee injuries. Of the male rowers, seven had rowed on the stroke side and six on the bow side. There were no significant differences in physical characteristics, muscle volumes, percent muscle volume to the total quadriceps femoris or Q-angle between the two sides (*p* = 0.183–0.993). All female rowers were scullers. The best record of ergometer all-out 2000 m rowing time was significantly faster in the male than in the female rowers (401 ± 12 s vs. 446 ± 17 s, *p* < 0.001). No significant difference was found (*p* = 0.058) in their rowing experience between sexes (male, 6.0 ± 2.1 yr; female, 4.6 ± 1.5 yr). They had won prizes at national college competitive meets in Japan, and some had participated in international competitive meets, including the Olympic Games. They belonged to the same university team, and conducted similar training programs during activities. They had been training daily, totaling approximately 17 hours/week, mainly by on-water rowing and partly by ergometer on the ground throughout a year. The training distance of on-water rowing was ~70 km/week on average. In addition, they conducted resistance exercises for the lower extremity twice/week (exercise program, power clean and back squat exercises; intensity, 4–12 repetition maximum load; volume, 4 sets of 3 reps–3 sets of 12 reps). The modality mainly consisted of multi-joint leg extensions, which are similar to the rowing motions. None of the control subjects had conducted conventional resistance training activities or high intensity sport activities at a high frequency (more than two days/week) for at least two years before the experiment. The study was approved by the Ethics Committee on Human Research of Waseda University, and done in accordance with Declaration of Helsinki. The subjects were informed of the purpose and risks of the study and provided written informed consent before the experiment.

### Magnetic resonance (MR) imaging measurement

**Fig 1:**
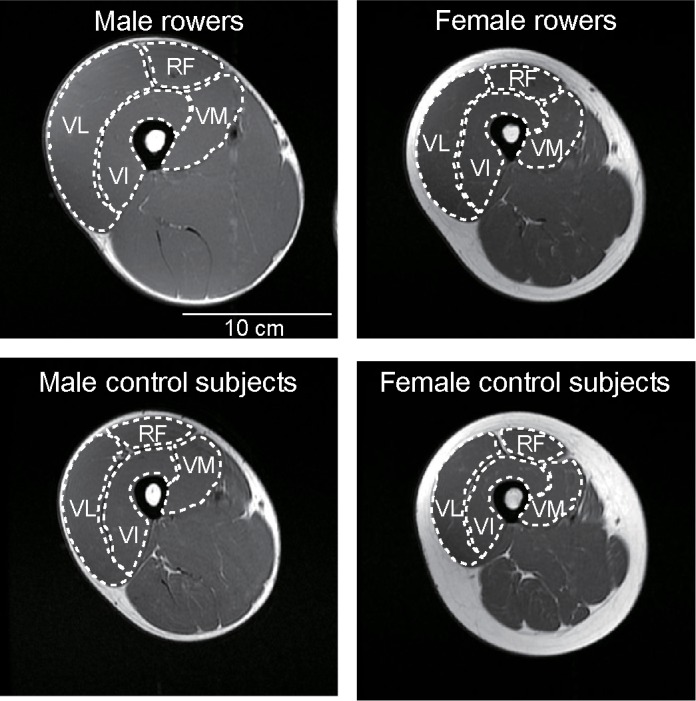
Examples of T1-weighted magnetic resonance images of the mid-thigh. The outline of individual muscles of the quadriceps is indicated by white dotted lines. VM, vastus medialis; VL, vastus lateralis; VI, vastus intermedius; RF, rectus femoris.

Using an MR scanner (Signa 1.5T; GE Healthcare, USA), a series of T1-weighted MR images (echo time: 10 ms, repetition time: 520 ms, matrix: 256 × 192, field of view: 24 cm, slice thickness: 1 cm) of the whole right thigh was acquired after the subject had been lying supine for at least 20 minutes [14]. The number of slices needed to cover the entire quadriceps femoris was 40 ± 3. All subjects were instructed to refrain from drinking alcohol the day before MR recordings. The subjects lay supine with their legs fully extended and muscles relaxed during MR recordings. From the MR images, the outlines of VL, VM, VI and RF were manually digitized, and the ACSAs of the muscles were determined (Fig 1) using ImageJ software (National Institute of Health, USA). Care was taken to exclude visible adipose and connective tissue incursions. Each image was digitized two times without knowledge of the Q-angle, and the mean values were used for further analysis. The CV, intraclass correlation coefficient type 1.2 (ICC [1,2]) and typical error [15] for the two measurements were 1.1%, >0.999 and 0.16 cm^2^, respectively. Muscle volume for each component muscle was determined by summing the ACSA-by-slice-thickness products. Because the volume of each muscle was significantly positively correlated with the body mass (r = 0.783–0.843, *p* < 0.001 for all muscles) and body mass was significantly greater in the rowers than in the controls in each sex (*p* = 0.001–0.002), the muscle volume was normalized to body mass (normalized volume). The percentage of each muscle volume to the total quadriceps femoris was also calculated.

### Q-angle determination

The Q-angle was measured in the right leg during quiet standing [9] by a different examiner from the one who analyzed the MR images, using a handmade goniometer made from a protractor and elastic string. The subjects stood with the knees fully extended, and the feet in a parallel position, with hand support as needed. The Q-angle was manually measured as the angle between the line connecting the anterior superior iliac spine with the center of the patella and the line connecting the center of the patella and the tibial tubercle [9]. The CV, ICC (1,1) and typical error of the measurement between days were 13.3%, 0.781 and 2.4°, respectively.

### Statistical analysis

Data are presented as means ± SDs. A three-way analysis of variance (ANOVA) with two between-group factors (rowing experience [rowers and controls], sex [male and female]) and one within-group factor (muscle [VM, VL, VI and RF]) was used for normalized volume to the body mass and percent volume of individual muscle to the total quadriceps femoris. A two-way ANOVA was performed to determine whether Q-angle differed between the rowers and controls or males and females. When a significant interaction was detected, follow-up ANOVAs with Bonferroni multiple-comparison tests were performed. The relationship between Q-angle and percentage of each muscle volume to the total quadriceps femoris was tested using Pearson’s product moment correlation coefficient. To investigate the magnitude of the difference in the normalized volumes, percent volume of each muscle to the total quadriceps femoris and Q-angle, Cohen’s d (between-subject designs, [16]) and 95% confidence interval (CI) of the difference were calculated. The significance level was set at *p* < 0.050. All the analyses were performed with SPSS version 22 (IBM, USA).

## Results

### Muscle volume

**Fig 2:**
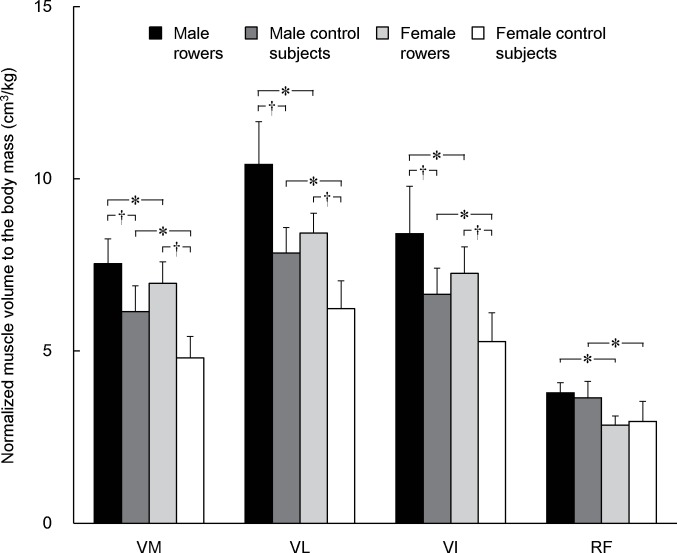
Muscle volume of each muscle normalized to body mass. * indicates a significant difference between male and female rowers or between male and female control subjects. † indicates a significant difference between rowers and control subjects within each sex. VM, vastus medialis; VL, vastus lateralis; VI, vastus intermedius; RF, rectus femoris.

An interaction of rowing experience × sex × muscle (*p* = 0.029) was significant for the normalized muscle volume to the body mass. The rowers had significantly greater normalized volumes of the vasti than controls, whereas no difference was found for RF between the rowers and controls (Fig 2, Table 1). Regardless of rowing experience, males had significantly greater normalized volumes than females in all muscles.

**Table 1:** Statistical results of the difference between rowers and untrained control subjects, and between males and females.

		P value	Cohen's d	95% confidence interval		
Normalized volume: Rowers vs. Controls						
Male	VM	< 0.001	1.89	0.91	to	1.91
	VL	< 0.001	2.62	1.95	to	3.21
	VI	< 0.001	1.65	1.08	to	2.49
	RF	0.372	0.36	-0.18	to	0.47
Female	VM	< 0.001	3.46	1.67	to	2.67
	VL	< 0.001	3.07	1.56	to	2.82
	VI	< 0.001	2.46	1.28	to	2.68
	RF	0.590	0.23	-0.41	to	0.24
Normalized volume: Males vs. Females						
Rowers	VM	0.034	0.85	0.04	to	1.11
	VL	< 0.001	2.07	1.34	to	2.68
	VI	0.003	1.04	0.41	to	1.90
	RF	< 0.001	3.38	0.60	to	1.28
Controls	VM	< 0.001	1.94	0.87	to	1.80
	VL	< 0.001	2.10	1.03	to	2.20
	VI	< 0.001	1.72	0.69	to	2.00
	RF	< 0.001	1.29	0.41	to	1.01
Percent muscle volume: Rowers vs. Controls						
Male	VM	0.668	0.16	-1.44	to	0.93
	VL	0.005	1.08	0.68	to	3.70
	VI	0.554	0.23	-1.06	to	1.95
	RF	< 0.001	1.65	-3.60	to	-1.16
Female	VM	< 0.001	1.44	1.20	to	3.57
	VL	0.370	0.32	-0.83	to	2.19
	VI	0.160	0.52	-0.43	to	2.58
	RF	< 0.001	2.25	-5.34	to	-2.90
Percent muscle volume: Males vs. Females						
Rowers	VM	0.001	1.62	-3.57	to	-1.05
	VL	0.071	0.78	-0.13	to	3.09
	VI	0.456	0.27	-2.20	to	1.00
	RF	0.032	1.19	0.13	to	2.72
Controls	VM	0.545	0.19	-0.77	to	1.44
	VL	0.960	0.01	-1.44	to	1.37
	VI	0.973	0.01	-1.38	to	1.43
	RF	0.577	0.16	-1.45	to	0.82

P value is the result of Bonferroni-multiple comparison test after a three-way analysis of variance. VM, vastus medialis; VL, vastus lateralis; VI, vastus intermedius; RF, rectus femoris.

### Muscle volume constituents

**Fig 3:**
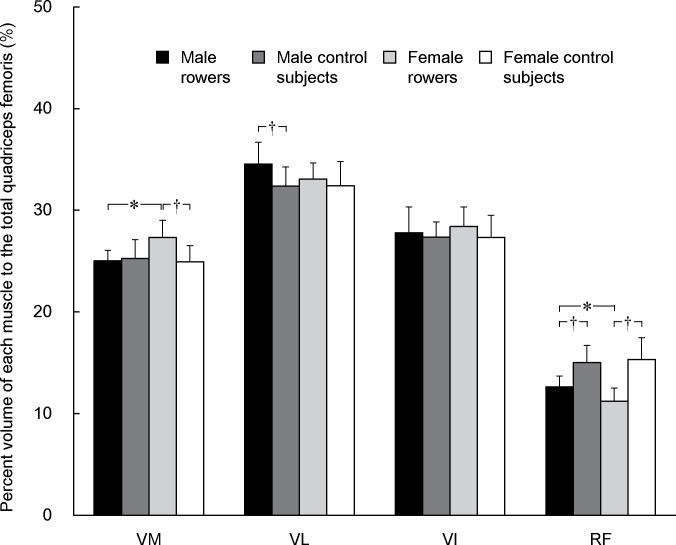
The percent muscle volume of each muscle to the total quadriceps femoris. * indicates a significant difference between male and female rowers. † indicates a significant difference between rowers and control subjects within each sex. VM, vastus medialis; VL, vastus lateralis; VI, vastus intermedius; RF, rectus femoris.

The three-way ANOVA revealed a significant rowing experience × sex × muscle interaction (*p* = 0.019) for the percentage of each muscle volume to the total quadriceps femoris volume. The percent VM volume in the female rowers was significantly higher than those of female controls and male rowers, and that of VL in the male rowers was significantly higher than that of male controls (Fig 3, Table 1). In contrast, the percentage of RF was significantly lower in the rowers than in the controls and that of the female rowers was significantly lower than that of the male rowers. The percentage of muscle volume did not differ significantly between the male and female controls for any muscles.

### Q-angle

There was a significant main effect of sex (*p* < 0.001) without a main effect of rowing experience (*p* = 0.862) or their interaction (*p* = 0.901) on the Q-angle. The Q-angles were significantly larger in the females (rowers, 19 ± 4°; controls, 18 ± 3°) than males (rowers, 14 ± 5°; controls, 14 ± 4°). Cohen’s d and 95%CI of the difference were as follows: male vs. female rowers, 0.91 and -7 to -1°; male vs. female untrained controls, 1.15 and -6 to -1°; male rowers vs. male controls, 0.01 and -3 to 3°; female rowers vs. female controls, 0.08 and -3 to 3°.

### Relationship between Q-angle and percent volume of each muscle

**Fig 4:**
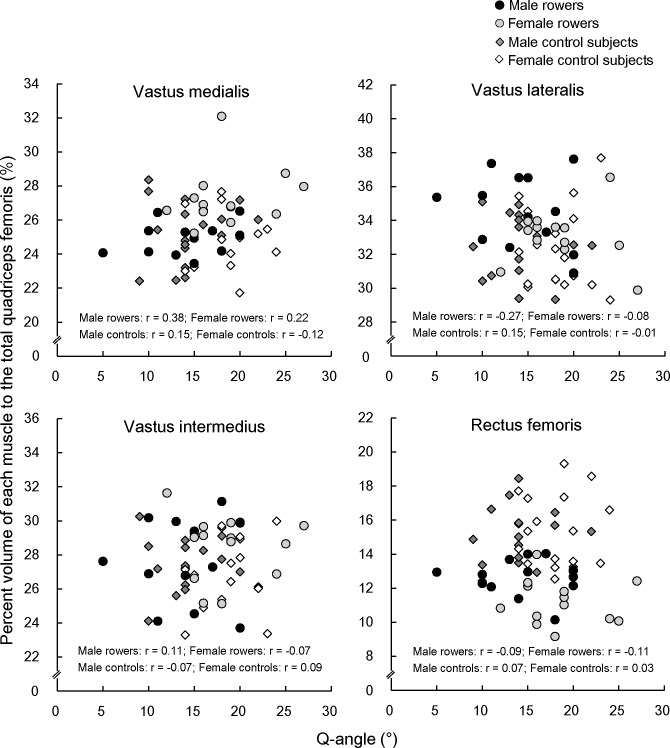
Relationship between Q-angle and percent muscle volume of each muscle to the total quadriceps femoris.

The relationships between Q-angle and the percent volume of each muscle to the total quadriceps femoris are presented in Fig 4. There were no significant correlations between Q-angle and percent muscle volume to the total quadriceps femoris in any muscles irrespective of rowing experience or sex (r = -0.27–0.38, *p* = 0.201–0.958).

## Discussion

The current study demonstrated that muscularity of the quadriceps femoris differs between male and female rowers but not between untrained male and female individuals, and rowers and untrained controls have different component characteristics of the quadriceps femoris in each sex. The percent VM volume to the total quadriceps femoris was higher in the female rowers than male rowers and female controls, and the percent VL volume was higher in the male rowers than male controls. The percent RF volume was lower in the rowers than controls in both sexes. In contrast, corresponding differences were not shown between the untrained male and female controls. Therefore, it can be said that the observed difference in the quadriceps femoris muscularity appears to be due to the sex-related specificity of hypertrophy by rowing training rather than a sex-related difference in inherited quantitative nature. Regarding the relationship between Q-angle and quadriceps femoris muscularity, Q-angle was not correlated with the percent volume to the total quadriceps femoris in any muscles in either rowers or controls. These findings suggest that Q-angle is not a major factor that affects the sex difference in the quadriceps femoris muscularity in rowers or controls.

The superior VM muscularity in female than male rowers was observed with a larger Q-angle. A large Q-angle results in a great lateral pull of the quadriceps femoris on the patella [9]. The VM, especially in the distal region, has been suggested to play an important role in resisting the lateral movement of the patella during dynamic weight-bearing conditions [17]. Based on these previous findings, there is a possibility that a rower with a large Q-angle needs to activate VM more compared with a rower with a small Q-angle so as to stabilize the patella when exerting a given quadriceps femoris force, leading to a gain of VM size. However, no relationship between percent VM volume and Q-angle was seen in each sex. Therefore, no evidence was found indicating that inter-individual variability in Q-angle substantially explains the sex difference in rowers’ muscularity.

The effect of event- or sex-related differences in the lower limb kinematics during rowing motions may also be related to the sex difference in the rowers’ muscularity. Due to differences in the number of oars and their positions (two symmetrically positioned oars in scull rowing and one oar at one side in sweep rowing), there may be a difference in the magnitude of hip external rotation during rowing motions among the events and/or sides (stroke and bow). In the current study, there were no significant differences in the right thigh muscularity or Q-angle of the male rowers between the two sides. In addition, it has been shown that muscle activation of the quadriceps femoris during squat and leg press was not affected by foot angles (30° forefoot abduction [i.e., hip external rotation] or no abduction) [3]. Thus, it is unlikely that the event-related differences in joint mechanics had a significant influence on the current findings. Regarding the sex differences in the joint kinematics, female rowers have been reported to tend to demonstrate a greater magnitude of femoral flexion (i.e., greater knee flexion) at the catch and through the stroke [18]. A previous training intervention study demonstrated that deep but not shallow squat training induced an increase in the front thigh ACSA in the distal region [19] where the proportional contribution of VM was large. This may suggest that exercises at relatively flexed knee joint angles induce preferential hypertrophy of VM after training. Collectively, it is possible that the sex-related difference in the knee joint kinematics existed for the rowers in the present study, which is associated with the sex difference in the quadriceps femoris muscularity in rowers.

Another possible factor for the sex difference in the muscularity is the difference in muscle fiber type composition. It has been reported that the proportion of type II fibers of VL is higher in men than in women [20]. Because the type II fibers show greater hypertrophy compared with type I fibers after resistance training [21], the difference in fiber type composition may be related to the preferential development of VL in male rowers. Regarding VM, to the best of our knowledge, there has been no direct comparison of fiber type composition between sexes, although no sex difference has been implied based on the median frequency of electromyogram during knee extensions [22]. Thus, it is difficult to conclude the sex difference in VM muscularity by a corresponding difference in its fiber type composition.

No sex difference was observed in the controls’ muscularity of the quadriceps femoris despite a sex difference in Q-angle. This result contradicts the previous description [23] that women have a smaller VM size in the distal region compared with men, but detailed comparison with the current data is difficult because they did not cite any concrete data. Moreover, the relations between Q-angle and percent volume of each muscle to the total quadriceps femoris were not significant in each sex. These findings suggest that knee alignment does not affect substantially the quadriceps femoris muscularity. This contradicts some previous findings in untrained healthy men [6,7]. The discrepancy may be related to some methodological differences in the evaluation of muscle size (ACSA at one region along the thigh in the previous studies) and knee alignment.

The percent RF volume to the total quadriceps femoris was lower in rowers than controls in both sexes, with the sex difference in Q-angle irrespective of rowing experience. Moreover, no correlation was observed between Q-angle and percent RF volume to the total quadriceps femoris. These results suggest that the inferiority of RF musculature in rowers is not related to the knee alignment. The findings of rowers’ RF musculature may result from the inter-muscle differences in magnitude of muscle activation during rowing motions. Muscle activation during on-water rowing normalized to those recorded during maximal voluntary isometric knee extension was over 40% in VL and VM, but less than 20% in RF in 5 male and 4 female rowers [24]. Considering a previous finding that the muscle activation of RF during daily life was less than 30% of maximal voluntary contraction [25], the magnitude of RF activation during rowing is likely to be insufficient to induce significant hypertrophy of the muscle. In contrast, it is possible that preferential activation of the vasti compared to RF during rowing motions is related to the greater normalized volumes of the vasti in rowers than in untrained subjects. The lower magnitude of RF activation during rowing may be related to the fact that rowing motions of the lower extremity mainly consist of multi-joint leg extensions. Muscle activation of RF during multi-joint leg extension was observed to be lower than that during single-joint knee extension exercises [3], likely resulting from the hip extension being involved in the multi-joint leg extensions [26]. Because RF is a biarticular muscle and thus its contraction produces hip flexion as well as knee extension moment, several inhibitory mechanisms for RF activation might be involved in multi-joint leg extensions.

Some limitations may be involved in the current study. The relatively low repeatability of Q-angle measurements may have diminished the magnitude of association between the quadriceps femoris muscularity and Q-angle. In contrast, if we determine the smallest worthwhile difference of Q-angle as 0.2 of between-subject SD [27], the value was approximately 1°. This is almost the same of 95% confidence limits of the difference between sexes: 95% CIs are unlikely to include the region which shows a trivial sex difference. Thus, it can be said that the substantial sex difference in Q-angle existed in the present study. Moreover, it remains unclear whether or not other types of training can induce similar hypertrophic response to those seen in the current study. Melnyk et al. [28] observed an increase in ACSA of the total quadriceps femoris in the distal region where the proportional contribution of VM was large but not in the proximal region after single-joint knee extension training in untrained women. In contrast, the ACSA increased in both distal and proximal regions in untrained men after the same training program as women [28]. Considering the current findings and Melnyk et al. [28], training-induced preferential VM hypertrophy might be occurred in women irrespective of its motion. In addition, our previous studies showed that experienced male cyclists had preferential development of VL compared with untrained male controls [12], which is in line with the present male rowers. These results might suggest that other types of training as well as rowing training induce muscle-specific adaptation of the quadriceps femoris.

## Conclusions

The current study revealed that well-trained rowers but not untrained individuals have sex-related (superior VM muscularity in females and VL in males) quadriceps femoris muscularity. No association was observed between Q-angle and percent muscle volume in any muscles regardless of rowing experience or sex. Therefore, our findings suggest that the difference in the rowers’ muscularity results from the sex-related specificity in hypertrophic response induced by rowing training, but the knee alignment is not a major determinant of the quadriceps femoris muscularity in either well-trained or untrained individuals.

## Supporting information

S1 Raw Data Physical characteristics and muscle volume of each subject. (XLSX)
